# Comparison of 3D Scanning *Versus* 2D Photography for the Identification of Facial Soft-Tissue Landmarks

**DOI:** 10.2174/1874210601812010061

**Published:** 2018-01-31

**Authors:** T. Zogheib, R. Jacobs, M. M. Bornstein, J. O. Agbaje, D. Anumendem, Y. Klazen, C. Politis

**Affiliations:** 1 OMFS-IMPATH Research Group, Department of Imaging and Pathology, Faculty of Medicine, University of Leuven and Oral and Maxillofacial Surgery, University Hospitals Leuven, Leuven, Belgium; 2 Applied Oral Sciences, Faculty of Dentistry, The University of Hong Kong, Prince Philip Dental Hospital, Sai Ying Pun, Hong Kong SAR, China; 3 Centre for Educational Effectiveness and Evaluation, Catholic University of Leuven, Leuven, Belgium; 4 SGS Life Science Services, Mechelen, Belgium

**Keywords:** 3D scanner, Face, 2D Photography, Soft-tissue analysis, Orthodontic and orthognathic treatments, Smile analysis

## Abstract

**Background:**

Three dimensional facial scanning is an innovation that provides opportunity for digital data acquisition, smile analysis and communication of treatment plan and outcome with patients.

**Objectives:**

To assess the applicability of 3D facial scanning as compared to 2D clinical photography.

**Materials & Methods:**

Sample consisted of thirty Caucasians aged between 25 and 50 years old, without any dentofacial deformities. Fifteen soft-tissue facial landmarks were identified twice by 3 observers on 2D and 3D images of the 30 subjects. Five linear proportions and nine angular measurements were established in the orbital, nasal and oral regions. These data were compared to anthropometric norms of young Caucasians. Furthermore, a questionnaire was completed by 14 other observers, according to their personal judgment of the 2D and 3D images.

**Results:**

Quantitatively, proportions linking the three facial regions in 3D were closer to the clinical standard (for 2D 3.3% and for 3D 1.8% error rate). Qualitatively, in 67% of the cases, observers were as confident about 3D as they were about 2D. Intra-observer Correlation Coefficient (ICC) revealed a better agreement between observers in 3D for the questions related to facial form, lip step and chin posture.

**Conclusion:**

The laser facial scanning could be a useful and reliable tool to analyze the circumoral region for orthodontic and orthognathic treatments as well as for plastic surgery planning and outcome.

## 1 INTRODUCTION

Over the past decade, noninvasive imaging techniques such as laser scanning have progressively gained popularity for oral and maxillofacial soft-tissue surface analysis [1, 2]. This is mainly due to its non-invasive and non-ionizing properties. This novel 3D technology, also known as facial scanning, gives a realistic representation of the head and face of the patient, which can be further used to analyze maxillofacial deformities, evaluate surgical outcomes, and assist in the diagnostic process in orthodontics, treatment planning, and follow-up [3, 4]. Recently, a growing interest for the use of facial scanning technology has also been manifested in prosthodontics and implant dentistry to optimize functional and esthetic outcomes [5].

Laser scanning relies on the simultaneous work of digital cameras and a low-intensity laser beam (below 0.00008 W) that has been shown to pose no risk to the patient [6, 7]. The low-intensity laser beam captures the surface texture while the digital cameras record the colors of the scanned surface. The laser scanning system, being a non-contact process, minimizes patient discomfort and potential contamination. In addition, and as far as soft-tissue landmarks are concerned, this technique avoids distortion of measurements due to pressure-related surface changes by direct contact of the scanning device [1, 8].

Several studies have investigated the accuracy and precision of the laser scanning for primarily qualitative but also quantitative three-dimensional orofacial structures [9, 10]. Yet, a true comparative study between the diagnostic gain of 2D facial pictures and 3D facial scans as assessed by quantitative measures and qualitative observations has not yet been carried out. Thus, the purpose of this study was to evaluate how quantitative measures and qualitative analyses on conventional facial 2D pictures are comparable to measurements on 3D reconstructed models of human faces using facial scanning technology.

## 2 MATERIALS AND METHODS

### 2.1 Study Design

For this study, 2D and 3D pictures were taken from 30 Caucasian volunteering subjects, working at the Department of Oral and Maxillofacial Surgery, Imaging and Pathology. The sample consisted of 16 women and 14 men; aged between 25 and 50 years (mean age of 30 years). The subjects had no history of dentofacial trauma or congenital syndromes causing facial malformations. No other exclusion criteria were applied. Ethical approval for this study was obtained from the Medical Ethical Committee of the University Hospitals of the Catholic University of Leuven (B322201316317). Additionally, written and verbal explanations were given to the eligible subjects, and all gave their informed consent to participate prior to commencement of the study. A power analysis was done to estimate the number of subjects required for this study. The results indicate that with 30 subjects included, all having 2D and 3D measurements, one will be able to detect a difference as small as 10% with a power of at least 80% for a standard deviation of 10 to 15%.

The 2D photographs and 3D facial scans were all taken by the same operator (TZ). The 2D photographs were captured with a Minolta 5 megapixel camera (Dimage 7i, Konica Minolta, Osaka, Japan), while the 3D facial scans were taken using the Proface feature of the ProMax^®^ 3D Mid (Planmeca, Helsinki, Finland).

**Fig. (1):**
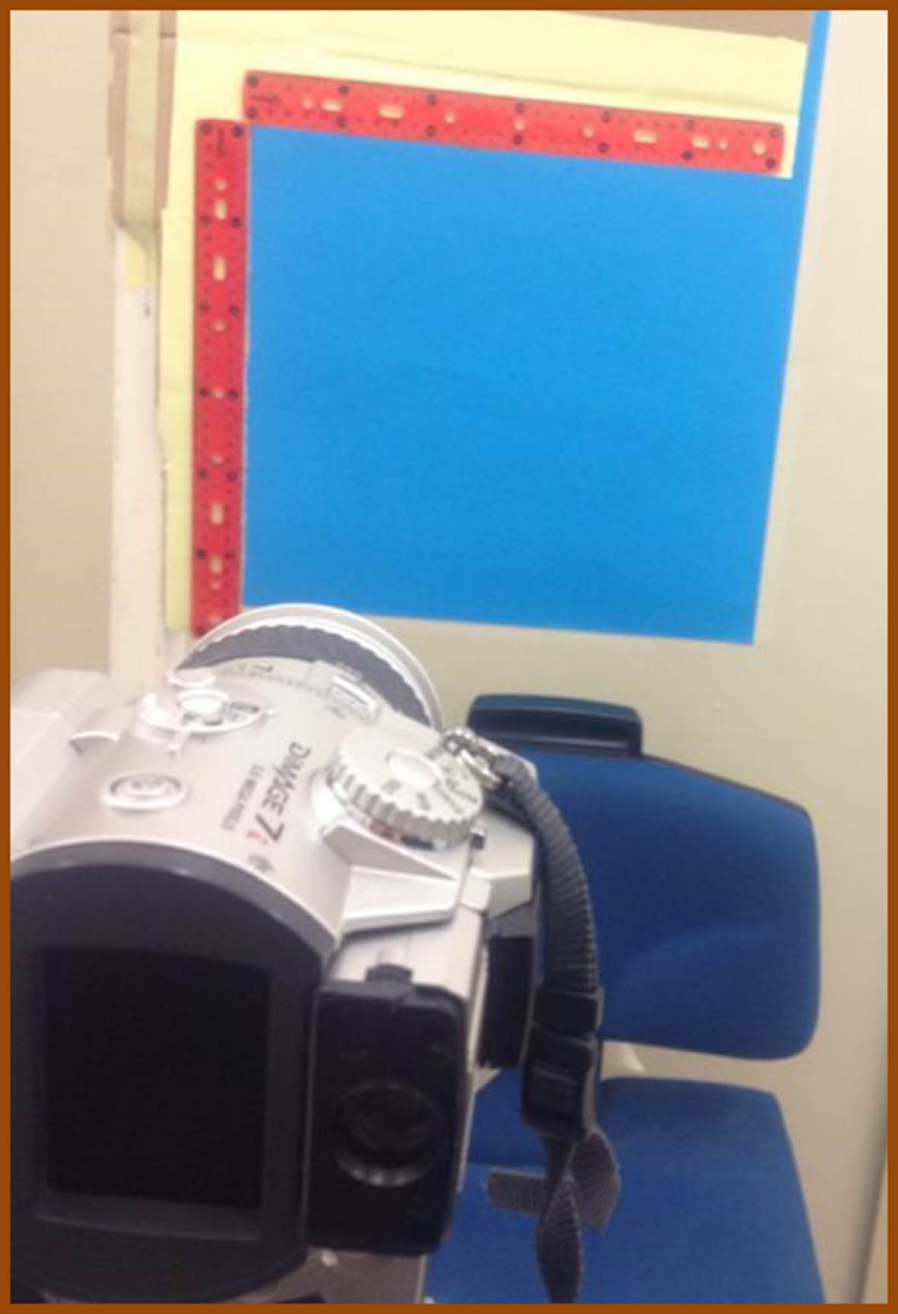
Set-up for 2D pictures.

The 2D images were taken in normal daylight. Three different 2D pictures were made under standardized conditions in a portrait mode: one smiling (frontal) and two non-smiling (one frontal and one lateral of the right profile) were taken to simulate pictures taken for routine diagnostic procedures in orthodontics and for orthognathic surgical treatment planning [11] Subjects were photographed sitting upright; head in the natural posture with the Frankfort Horizontal plane (FH plane) parallel to the floor, looking at an image of themselves in a mirror at eye level behind the camera. The FH plane was defined as the plane passing through the upper borders of each auditory canal or external auditory meatus (porion), and through the inferior margin of the left orbit [12-14]. The photographs were standardized by using a precise reproducible set-up of a tripod, camera, and chair for the subject leaving a distance of 1.5 meters between the patient and the camera lens. An L-shaped ruler was placed on the upper right side of the subjects, as a calibration tool to compensate for the magnification of the image. A blue background was used to improve contrast (Fig. **1**). Closed or red eyes, shadows on the face and wearing glasses were avoided.

**Fig. (2):**
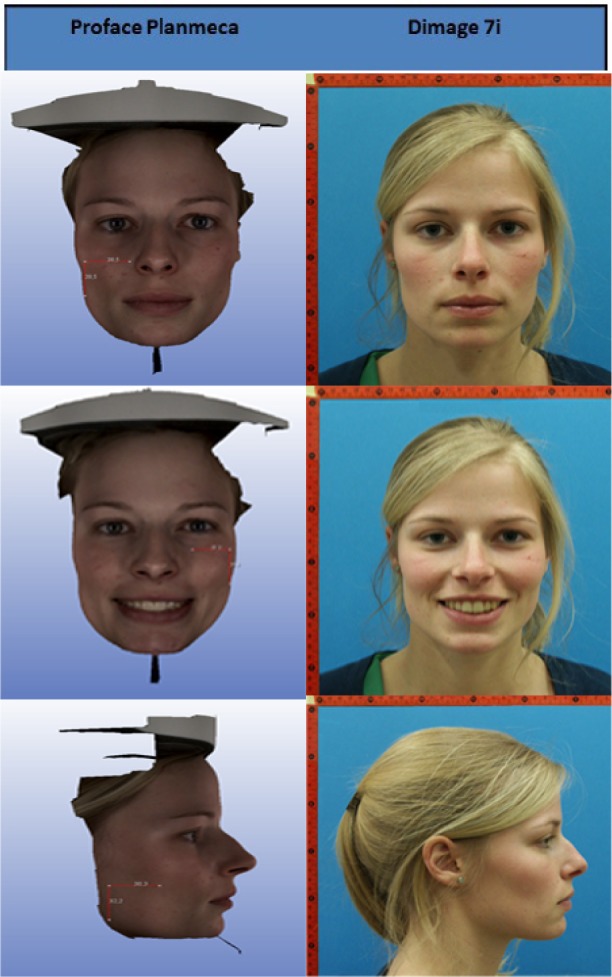
3D facial scans and 2D pictures of one of the volunteers placed next to each other for comparison.

Two 3D facial scans (one smiling and one non-smiling) were acquired for each subject using the ProMax^®^ 3D (Fig. **2**). All subjects were asked to look straight ahead, keeping their heads parallel to the FH plane, and to avoid blinking their eyes or moving during the scanning process to prevent artifacts in the final image. The subjects also had to clear their head and neck region from any interfering jewelry, clothing elements or glasses as these factors can trigger artifacts by intensely reflecting the light during data recording [1].

The acquired 2D (TIFF-file) and 3D images (OBJ file) were afterwards transferred to a computer (DELL OPTIPLEX 7010, screen model number P2312Ht, DELL, Texas, United States) for further analysis on a standard diagnostic display (Dell 17 inch monitor).

### 2.2 Quantitative Assessment of Facial Morphology: Accuracy Assessment

**Fig. (3):**
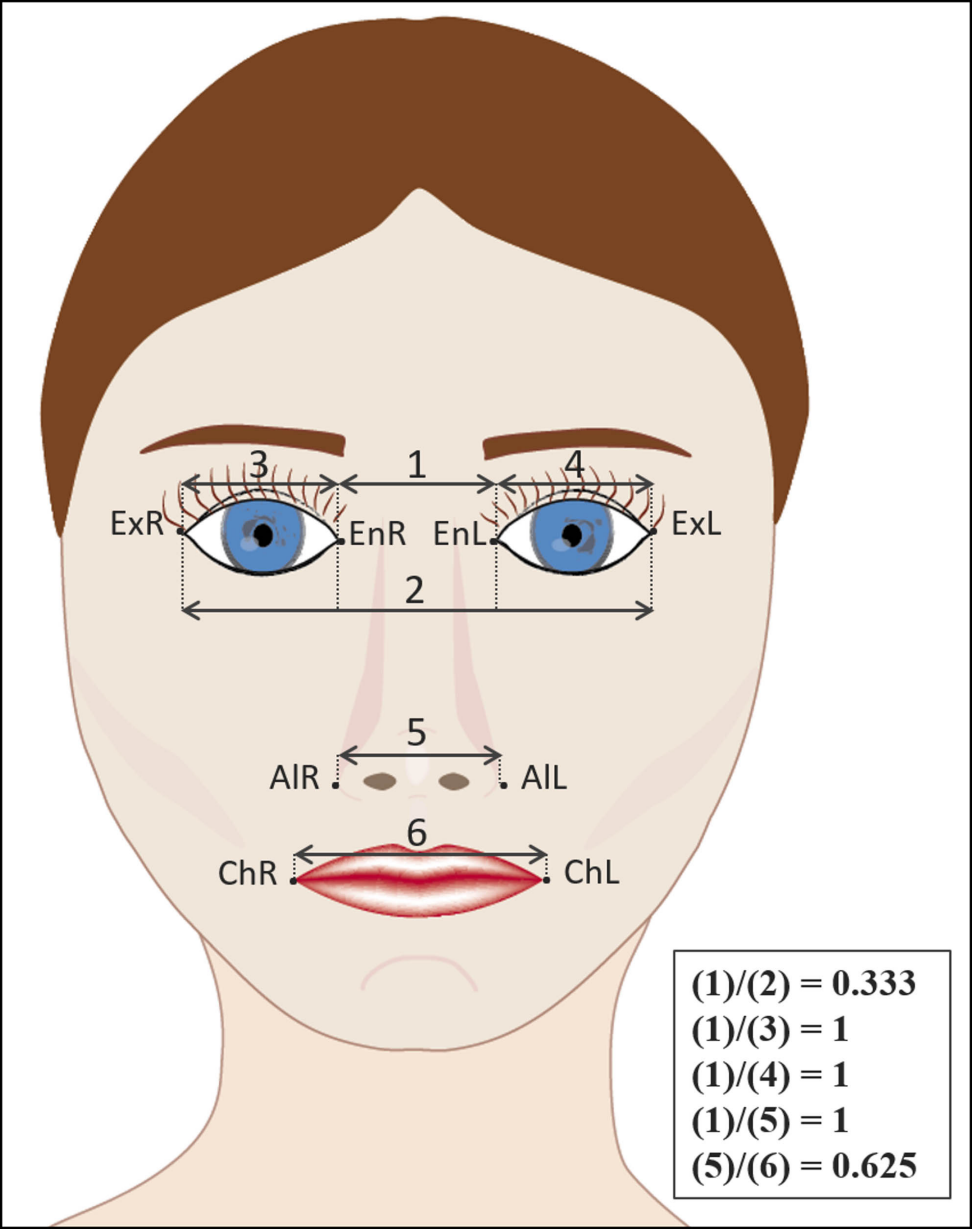
Facial landmarks and linear ratios as evaluated in the frontal view (for details regarding the abbreviations see Table **1**).

**Fig. (4):**
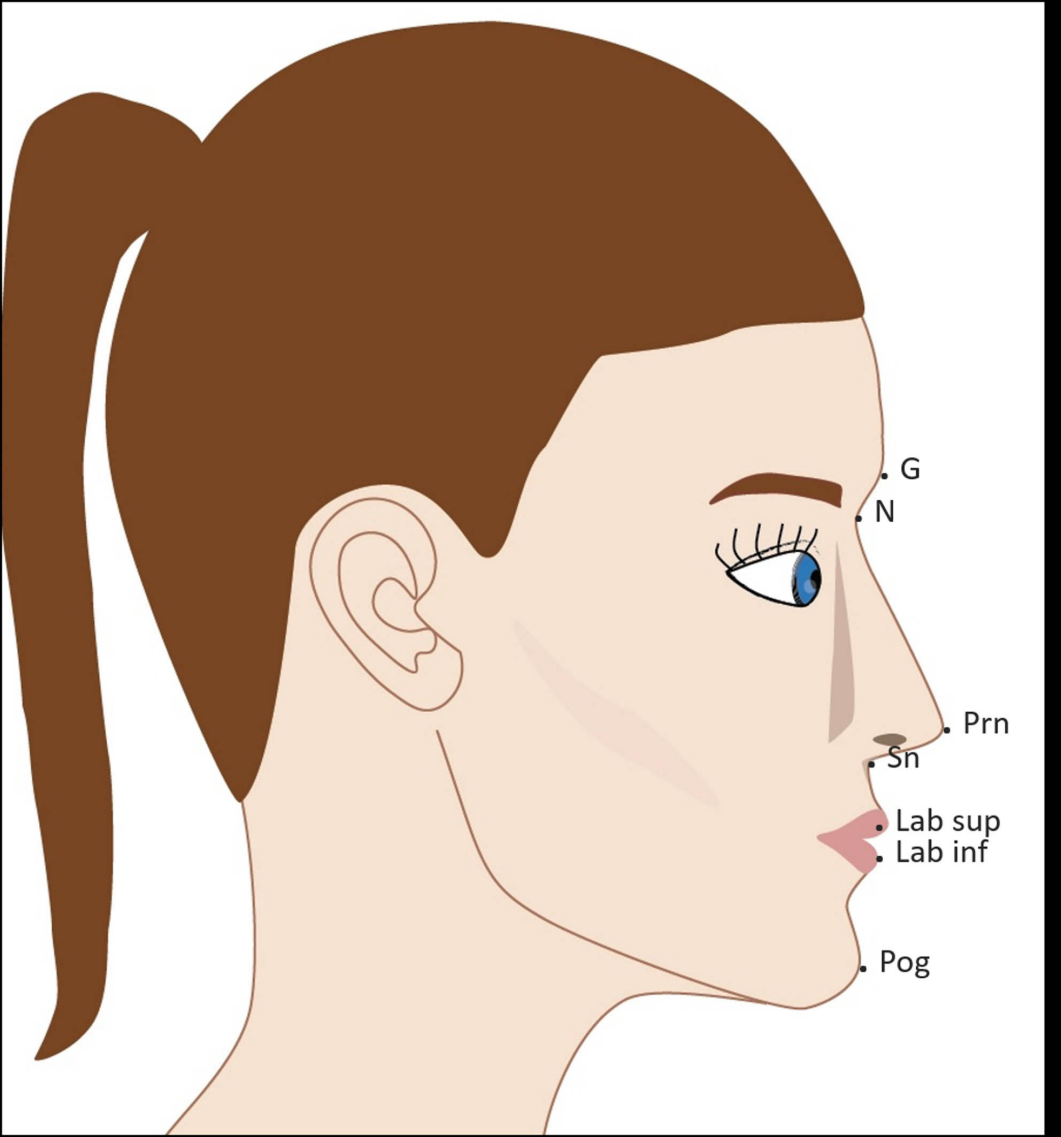
Facial landmarks as evaluated in the lateral view (for details regarding the abbreviations see Table **1**).

A total of 15 facial landmarks - 7 single and 4 paired (Table **1**; Figs. **3** and **4**) - were identified twice on each of the 2D and 3D images by three qualified dental observers (all specialists in dento-maxillofacial radiology) after an initial calibration session for identification of facial landmarks. To place the landmarks on the 2D images, Adobe Photoshop Creative Cloud (Adobe Systems Incorporated, San Jose, CA, USA) was used. Maxilim (Medicim, Sint-Niklaas, Belgium) software was used for landmark identification on the 3D images.

Landmark identification on 2D and 3D were repeated by all three observers after an interval of 2 weeks. All landmarks were placed on the images using the same computer under identical lighting conditions. During the observations, a chart with the description of each landmark point was provided for assistance [8, 15-20]. Furthermore, observations were followed by a supervisor (TZ) to assure standardization of the readings and assist in the use of the software program without helping directly in the landmark identification. The linear (mm) and angular (degrees) measurements were then assessed on the 2D and 3D images (Fig. **1** and Table **2**, respectively). Recorded linear measurements from each subject’s 2D facial picture and 3D facial scan (n=30) were compared to measurements of the same facial anthropometric values directly assessed on the subject’s face with a digital caliper. This allowed for accuracy and reliability assessment.

### 2.3 Qualitative Assessment of Facial Morphology: Observers’ Questionnaire

Apart from quantitative measures using linear and angular values (Tables **2** and **3**) based on facial landmarks, a qualitative evaluation of the 2D and 3D facial photographs was done (Table **4**). For this purpose, 2D and 3D images were displayed next to each other, and were analyzed by an additional 14 qualified observers (all dentists from the University Clinics of Leuven) who also indicated their confidence level for each given answer.

### 2.4 Statistical Analysis

All statistics were carried out in SPSS (IBM SPSS Statistics for Windows, version 22.0, IBM Corp. Released 2013, Armonk, NY, USA) and in SAS (version 9.4, SAS Institute Inc., Cary, NC, USA). The significance level was set at *p*-value < 0.05. Pearson’s correlation coefficient was used to determine the inter- and intra-observer reliability of the landmark identifications. After checking for normality of the numerical data distribution with Shapiro-Wilk test, ANOVA parametric analysis was used to assess the landmark identification by comparing ratios and angles from 2D photographs and 3D facial scans to the corresponding clinical standards. Wilcoxon signed rank test was performed on the confidence scores given per observer for each question and image, to define the total confidence level in 3D compared to the one in 2D. Intra-observer Correlation Coefficient (ICC) was used to assess the reliability of the observers’ replies, per question per case.

## 3 RESULTS

### 3.1 Quantitative Assessment of Facial Morphology: Accuracy Assessment

#### 3.1.1 Linear Measurements

Quantitatively, proportions linking the three facial regions in 3D were closer to the clinical standard than proportions in 2D (for 2D 3.3% error on average and for 3D 1.8% error rate on average). The distribution of linear ratios showed that 3D values were closer to the clinical standard in the ratio concerning endocanthion distance relatively to nose width at alare region (EnR; EnL) / (AlR; AlL). The estimate difference between 3D and clinical standard was -0.08 with *p*-value <0.001, whereas between 2D and clinical standard this value was -0.20 with *p*-value <0.001). The ratio values of nose width at the alare region relatively to mouth width (AlR; AlL) / (ChR; ChL) were also closer to and non-significantly different from the clinical standard in 3D (estimate difference between of 0.02; *p*-value of 0.12), whereas between 2D and the clinical standard it was 0.04 with a *p*-value of 0.001. The most significant differences in 3D and 2D compared to the standard values were seen in eyes measurements relating each eye width to the endocanthion distance (EnR; ExR) / (EnR; EnL) and (EnL; ExL) / (EnR; EnL).

#### 3.1.2 Angular Measurements

Concerning the angular measurements, 3D values were insignificantly different from 2D in 8 of the 9 cases and both followed the same variation pattern relatively to the clinical standard. Most of the angles did not significantly differ. Yet, there was a tendency for more accurate point determination when comparing 3D facial measures to the clinical reference of the involved subnasale, labiale superior, labiale inferior, and pogonion.

For the quantitative assessment of the facial anthropomorphic points, Pearson’s correlation coefficient was 0.98 for all observers when comparing 2D and 3D facial images to the clinical reference (direct facial measures). The intra- and inter-observer reproducibility of landmarks was high (< 0.5 mm SD) for all linear ratios in 2D and 3D images.

#### 3.1.3 Qualitative Assessment of Facial Morphology: Observers’ Questionnaire

Qualitatively, Wilcoxon test showed that in 67% of the cases, the 14 observers for the subjective assessment of facial morphology were as confident about 3D reconstructions as they were about 2D images. In 17.8% of the cases, they were more confident about 2D and in 15.2% of the cases, they were more confident about 3D. ICC revealed a better agreement regarding 3D imaging between observers in the 3 questions related to the facial form, lip step and chin posture (ICC in 3D were 0.55, 0.76, and 0.73 respectively, while their corresponding ICC in 2D were 0.48, 0.74, and 0.60).

## 4 DISCUSSION

Laser scanning is a non-invasive, non-ionizing imaging technique, which captures the facial soft-tissues in three dimensions. The purpose of this study was to evaluate the clinical applicability of currently available 3D facial scan analysis by comparing 3D facial scans to 2D facial photographs. The study was done to mimic clinical orthodontic diagnostic analysis, where professionals rely on the clinical standard ratios and angles for their treatment planning. The use of mid-sagittal facial landmarks on the lateral pictures, and paired landmarks on the frontal ones, were selected according to Farkas *et al*. [21].

On one hand, our results showed that the ratios (EnR; EnL) / (AlR; AlL) and (AlR; AlL) / (ChR; ChL) - involving the intercanthal, nasal and oral widths, obtained from the 3D reconstructions were closer to the clinical standard values. These landmarks seem to be more reliable when assessed in 3D. The measurement of the width of the mouth (ChR; ChL) was considered to involve ones of the most accurate landmarks [22]. On the other hand, the most significant differences in 2D and 3D were seen for measurements concerning eye dimensions - (EnR; ExR) / (EnR; EnL) and (EnL; ExL) / (EnR; EnL). The observed variability in the subjects’ eyes could be due to the degree of persistence of the epicanthic fold after maturity [23, 24]. This variability could also be explained by the anatomical concavities of the orbital region that could modify the perception of the eyes. In fact, Abate *et al*. [25] found that, because of the 3D structure of the face, a direct lighting source can create strong shadows on the facial skin, leading to dissimilarities in appearance that are larger, if induced by illumination than by the morphological inter-individuals differences. This is also supported the study from Bowyer *et al*. [26] which demonstrated the projection errors of a 3D face on a 2D picture, along with the issues of 2D photography, namely lighting and pose variations, which may cause landmarks identification difficulties.

Pearson’s correlation coefficient of 0.98 indicated a high inter-observer reliability in the placement of landmarks (correlation coefficient reliable if > 0.8 [17]).

Concerning the angular measurements, 3D was at least as reliable as 2D in most of the cases. The angles in the midsagittal region around the circumoral area involving subnasale, labiale superior, labiale inferior and pogonion were reliable on the facial scans. This result is in agreement with data obtained by Aung *et al*. [10] and Toma *et al*. [15], where they proved labiale superior, as well as other midsagittal landmarks in this facial area to be very reliable on 3D images. The identification of mid-sagittal facial landmarks (used for angular measurements) seemed to be more precise than the identification of paired landmarks (used for linear ratios), which agrees with the results of Plooij *et al*. [17]. In their study however, facial landmarks were identified on facial scans done by a stereophotogrammetric device rather than with a laser scanning machine.

The three qualified observers participating in the quantitative part of this study were not orthodontists but they were all calibrated to place the specific landmarks on 2D and 3D images for this research. According to Gwilliam *et al*. [16], even experts such as orthodontists have limited experience in placing facial soft tissue landmarks on 3D computer images. They are rather familiar with tracing lateral cephalograms and analyzing 2D pictures. However, further research comparing experienced versus non-experienced clinicians would be required.

In contrast to 2D photogrammetry, the soft tissue landmarks do not need to be indicated on the patient’s face prior to capturing the 3D image [17, 27]. In fact, computer software, such as Maxilim, offer a virtual placement of landmarks on a subject’s face with the possibility of modifying or inserting additional points based on the needs of the respective analysis [1]. The option of rotating and magnifying the scanned faces also plays an important role in localizing the landmarks on the computer screen [17, 28].

In the questionnaire of the present study, the slightly better agreement between the 14 observers in 2D (17.7% *versus* 15.2% in 3D) could be due to the relative novelty of the 3D imaging. This means some observers are still not well acquainted with the analysis of such reconstructions. However, these observers still had substantial confidence in the visualized 3D images (as much confidence as in the 2D pictures, *e.g*. 67% of the cases).

The ICC, used to assess the rating reliability, compared the variability of different observers of the same case to the total variation across all observers and cases. The fact that the facial form, lip step and chin posture were better seen on 3D images emphasizes that the 3D imaging with laser-scanning techniques could be used as a tool to detect deformities in the oral area and assess changes in facial morphology as a result of orthodontic treatment, surgery, and facial growth [29]. Furthermore, this gives a considerable advantage to the facial laser scanning technique due to the importance of these anatomical features in orthodontics soft-tissue analysis. In fact, since the lips play a key role in determining the person’s smile, eating and speaking functions, the lip-chin area is meticulously evaluated before any eventual modification by orthodontic treatment or orthognathic surgery. The labial area is therefore one of the most important components of diagnosis and treatment planning [29]. The progressive switch to 3D image for soft tissue analysis might be justified when a precise documentation of long-term extra-oral follow-up is needed, especially when it is not mandatory or ethically suitable to use CBCT data [30].

When combined with a CBCT, the facial scan has the potential to visualize the bone-soft-tissue relationship, which is fundamental for soft tissue variables such as lip or chin thickness for diagnosis and treatment plan.

It has already been proven that laser scanning is at least as precise as the traditional manual methods for linear measurements [7]. One disadvantage may be the time necessary for a complete facial scan (between 8sec and 15sec), which is significantly higher than that necessary for stereophotogrammetry (1.5ms) [8, 31], possibly leading to distortions and motion artifacts during the scanning process [1, 2]. Therefore, this approach is not well-suited for people with limited cooperation such as agitated patients or children [1]. The presence of multiple facial subcutaneous muscles makes facial appearance instantaneously variable and dynamic, thus causing potential problems for linear or angular measurement if not captured quickly enough [32-35]. Another drawback of 3D imaging is the incomplete scan of the facial surfaces, particularly leaving out the hairline, ears and chin. An implication of this constraint in the field of view is a restricted scanned facial area to study, thus a limited applicability of linear ratios and angular measurements involving edge-landmarks such as trichion, porion, gnathion or menton.

## CONCLUSION

Based on the findings of the present study, the following conclusions can be made:

- In comparison to the classical 2D photography, 3D facial scanning seems to better illustrate the clinical standard measurements of the human face for the linear proportions linking the orbital, nasal and oral regions to each other, while being as good as 2D for angular measurements involving the lips and subnasale landmarks.
- Observers showed a substantial confidence in 3D reconstructions, and agreed better on the analysis of the lips and chin in 3D in comparison to 2D measurements.
- The laser facial scanning could therefore be a useful and reliable tool to analyze the circumoral region for orthodontic and orthognathic surgical diagnosis and treatment planning. In order to take clinical advantage of this new technology, 3D facial scanning could be combined with CBCT to measure the thickness of the perioral soft-tissues.

However, the problems of the restricted scanned facial area and the capturing speed need to be solved.

## Figures and Tables

**Table 1:** Facial soft-tissue landmarks (n = 15; 7 single and 4 paired) evaluated in the present study.

**Landmark**	**Definition**
Glabella (G)	The most prominent midpoint of the frontal bone, between the eyebrows.
Nasion (N)	The deepest point of the nasal bridge, on the intersection between the midline of the nasal root and the nasofrontal suture.
Endocanthion left (EnL) and right (EnR)	The point at the inner commissure of the left and right eye fissures.
Exocanthion left (ExL) and right (ExR)	The point at the outer commissure of the left and right eye fissures.
Pronasale (Prn)	The most protruded point of the apex nasi, identified in lateral view of the natural head position.
Subnasale (Sn)	The midpoint of the angle at columella base.
Alare left (AlL) and right (AlR)	The most lateral point on each of the alare contours.
Labiale superior (Lab sup)	The most prominent midpoint of the upper vermilion line.
Labiale inferior (Lab inf)	The most prominent midpoint of the lower vermilion line.
Cheilion left (ChL) and right (ChR)	The most lateral points of the labial commissures, joining the superior and inferior lips.
Pogonion (Pog)	The most anterior midpoint of the soft tissue of the chin.

**Table 2:** Linear measurements.

Linear ratios
(EnR -> EnL) / (ExR -> ExL):
(Distance between endocanthion right and endocanthion left)/(Distance between exocanthion right and exocanthion left)
(EnR -> ExR) / (EnR -> EnL):
(Distance between endocanthion right and exocanthion right)/(Distance between endocanthion right and endocanthion left)
(EnL -> ExL) / (EnR -> EnL):
(Distance between endocanthion left and exocanthion left)/(Distance between endocanthion right and endocanthion left)
(EnR -> EnL) / (AlR -> AlL):
(Distance between endocanthion right and endocanthion left)/(Distance between alare right and alare left)
(AlR -> AlL) / (ChR -> ChL):
(Distance between alare right and alare left)/(Distance between cheilion right and cheilion left)

**Table 3:** Angular measurements and corresponding clinical standard values from literature.

Angular Measurements	Clinical Standard
Holdaway’s harmony angle= N-Pog-Lab sup: Angle between the soft tissue nasion, pogonion and the most prominent point of the upper lip: labiale superior.	10°
Lab sup-G-Pog: Angle between the soft tissue labiale superior, glabella and pogonion.	6.3°
Lab inf-G-Pog: Angle between the soft tissue labiale inferior, glabella and pogonion.	3.3°
G-N-Prn: Angle between the soft tissue glabella, nasion and pronasale.	140.3°
Pog-Prn-N: Angle between the soft tissue pogonion, pronasale and nasion.	129.5°
Prn-N-Sn: Angle between the soft tissue pronasale, nasion and subnasale.	22.5°
Prn-N-Pog: Angle between the soft tissue pronasale, nasion and pogonion	27.5°
(N-Prn)/(G-Pog): Angle between the lines joining soft tissue nasion to pronasale and glabella to pogonion.	35°
(Sn-Lab sup)/(Pog-Lab inf): Angle between the lines joining soft tissue subnasale to labiale superior and pogonion to labiale inferior.	157.3°

**Table 4:** Questionnaire used for qualitative assessment by 14 independent observers (all dentists).

**Frontal ** **Images**	**1. Facial form **	Leptoprosopic
		Mesoprosopic
		Euryprosopic
	1'. How confident are you about your answer?*	
	**2. Corners of the mouth**	Higher than horizontal
		Horizontal
		Lower than horizontal
	2'. How confident are you about your answer?*	
**Profile** **Images**	**3. Nasolabial angle**	> 90°
		90°
		< 90°
	3'. How confident are you about your answer?*	
	**4. Lip closure**	Competent lips
		Incompetent lips
	4'. How confident are you about your answer?*	
	**5. Lip step**	Enlarged lip step
		Normal lip step
		Reverse lip step
	5'. How confident are you about your answer?*	
	**6. Chin posture**	Prominent
		Normal
		Recessed
	6'. How confident are you about your answer?*	
–	**7. To assess the facial soft tissues of a patient, which of the following modalities would you feel ** **more confident using and analyzing?**	2D photographs
		3D facial scans

*Please rate your confidence in ability to judge the displayed images on a scale of 1 to 3, where 1 means “Not confident at all” and 3 means “Very Confident.”

## LIST OF ABBREVIATIONS

2D: Two-dimensional
3D: Three-dimensional
CBCT: Cone Beam Computed Tomography
